# The Learning Curve of Gastric Intestinal Metaplasia Interpretation on the Images Obtained by Probe-Based Confocal Laser Endomicroscopy

**DOI:** 10.1155/2012/278045

**Published:** 2012-12-01

**Authors:** Rapat Pittayanon, Rungsun Rerknimitr, Naruemon Wisedopas, Suparat Khemnark, Kessarin Thanapirom, Pornpahn Thienchanachaiya, Nuttaporn Norrasetwanich, Kriangsak Charoensuk, Wiriyaporn Ridtitid, Sombat Treeprasertsuk, Pradermchai Kongkam, Pinit Kullavanijaya

**Affiliations:** ^1^Division of Gastroenterology, Department of Medicine, Chulalongkorn University, Bangkok 10330, Thailand; ^2^Department of Pathology, Chulalongkorn University, Bangkok 10330, Thailand

## Abstract

*Background*. Reading the results of gastric intestinal metaplasia (GIM) with probe-based confocal laser endomicroscopy (pCLE) by the expert was excellent. There is a lack of study on the learning curve for GIM interpretation. Therefore, we conducted a study to explore the learning curve in the beginners. *Material and Method*. Five GI fellows who had no experience in GIM interpretation had been trained with a set of 10 pCLE video clips of GIM and non-GIM until they were able to interpret correctly. Then they were asked to interpret another 80 video clips of GIM and non-GIM. The sensitivity, specificity, accuracy, PPV, NPV, and interobserver agreement on each session were analyzed. *Results*. Within 2 sessions, all beginners can achieve 80% accuracy with substantial to almost perfect level of interobserver agreement. The sensitivities and specificities among all interpreters were not different statistically. Four out of five interpreters can maintain their high quality of reading skill. *Conclusion*. After a short session of training on GIM interpretation of pCLE images, the beginners can achieve a high level of reading accuracy with at least substantial level of interobserver agreement. Once they achieve the high reading accuracy, almost all can maintain their high quality of reading skill.

## 1. Introduction

 Gastric cancer is the second leading cause of cancer death worldwide [1], and gastric intestinal metaplasia (GIM) is the precancerous lesion for intestinal type gastric cancer [2, 3]. The strategies which can detect precancerous and/or early cancerous transformation are very beneficial because only early gastric cancer can potentially be cured by endoscopic treatment. Probe-based confocal laser endomicroscope or pCLE is one of the useful equipments for GIM detection. The endoscopic criteria for GIM reading by pCLE were (1) villous-like gastric epithelium and (2) dark (no fluorescein uptake) goblet cells in the gastric columnar epithelium [4]. Previously our group reported the results of GIM detection and interpretation by pCLE as 94% in sensitivity, 85% in specificity, and 89% in accuracy [4]. However, these excellent results in pCLE interpretation were established by the expert. The study on learning curve by the beginners for the new type of image reading including GIM interpretation by pCLE is important for community practice. Therefore, we conducted a study to explore the learning curve pattern by the beginners.

## 2. Material and Method

### 2.1. Procedure and Data Collection

This study was conducted at the Division of Gastroenterology, Department of medicine, Chulalongkorn University. The protocol study was registered through ClinicalTrials.gov (NCT01491724) and approved by the Chulalongkorn University Institutional Review Board. Fifty patients with previous histologies confirmed as GIM underwent a surveillance gastroscopy with pCLE (Mauna Kea Technologies, Paris, France) performed by one endoscopist (RP) under a standard conscious sedation with intravenous midazolam (Cenexi SAS, Fontenay-sous-Bois, France) and meperidine (M&H manufacturing Co., Ltd., Samutprakarn, Thailand). For the best quality of pCLE images, 10 milligrams of hyoscine (Pharmaland (1982) Co., Ltd, Bangkok, Thailand) were given before the procedure to decrease the bowel movement.

After an intravenous injection of two and a half milliliter of 10% fluorescein (Novartis Pharmaceutical Corporation, Bangkok, Thailand), pCLE video clips in MPEG format with duration of 40–120 sec were obtained from GIM and non-GIM suspicious area. After histology confirmation of both GIM and non-GIM video clips, the experienced pCLE endoscopist (RP) selected 90 high quality pCLE video clips (clearly visualized gastric epithelium, vessel and goblet cell, and good image stability) for further interpretation by the beginners. Of those, 45 video clips corresponded to normal mucosa and the other 45 video clips represented GIM. All video clips were incorporated into a slideshow format (Microsoft PowerPoint 2003) with the play duration of 10–20 sec.

The beginners in this study were defined as first-year GI fellows who had no experience in pCLE images interpretation. Five eligible GI fellows (SK, KC, PT, NN, and KT) from Chulalongkorn University were recruited to the study. They were trained by the experienced endoscopist (RP) in the training class for 1 hour with an initial set of 10 pCLE video clips representing 5 GIM and 5 non-GIM. The criteria for GIM reading by pCLE were (1) villous-like gastric epithelium and (2) dark (no fluorescein uptake) goblet cells in the gastric columnar epithelium [4]. Then, they were provided with the same set of 10 video clips for self-practice until they were able to interpret all 10 video clips correctly. All video clips used for the learning set were not included in the study sets.

To assess the learning curve, the other 80 high quality pCLE video clips were divided into 4 sets of 20 video clips. All beginners interpreted the 4 sets of 20 pCLE video clips of GIM and non-GIM at 2-week interval. For each testing session, all interpreters were asked to sit in a class room and viewed the video clips displayed by a projector. Each video clip was played twice before the interpreters chose their answers (as GIM or non-GIM). After each session, their scores and the correct answers of video clips were not revealed to any readers. 

### 2.2. Statistical Analysis

The outcome in GIM interpretation by pCLE of each beginner was assessed for the sensitivity, specificity, accuracy, positive predictive value (PPV), and negative predictive value (NPV). For numerical variables, the results were expressed as a mean ± SD whereas other quantitative variables were shown in percentage. Fleiss' kappa (*κ*) was used to analyze the interobserver agreement among 5 beginners on those captured pCLE images. The value of kappa (*κ*) for agreement was graded as poor for 0.01 to 0.20, fair for 0.21 to 0.40, moderate for 0.41 to 0.60, substantial for 0.61 to 0.80, and almost perfect for 0.81 to 1.00. 

## 3. Results

**Figure 1:**
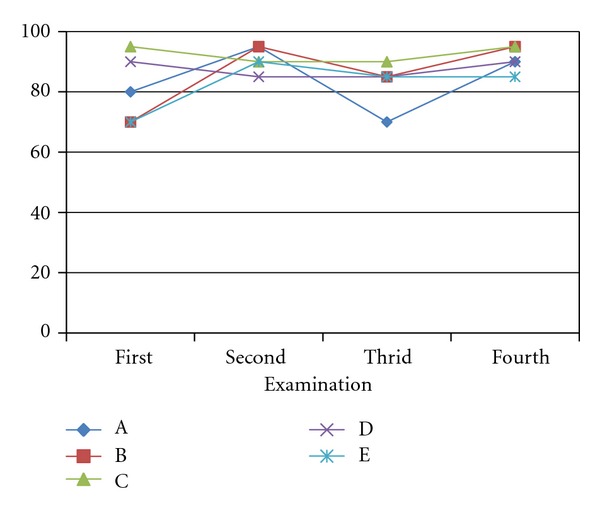
Accuracies of different beginners. A: beginner number 1, B: beginner number 2, C: beginner number 3, D: beginner number 4, E: beginner number 5.

From the first session, the overall sensitivity and specificity for GIM diagnosis of the 5 beginners were above 70% and gradually increased over time. The PPV was more than 90% on average, whereas NPV was lower at 75–85%. Among all interpreters, there were no differences in statistical analysis of all validity scores (Table 1). Additionally, one beginner achieved the 100% sensitivity, 91.6% specificity, and 95% accuracy within the first session. Within two sessions, all beginners were able to provide more than 80% in accuracy with substantial to almost perfect level of interobserver agreement (Table 2). Almost all of them (4 from 5) were able to maintain their good reading skill with 85–95% accuracy through the rest of study sessions (Figure 1).

## 4. Discussion

Confocal laser endomicroscope (CLE) is the latest novel endoscopic technology which has been commercially available since 2005 [9]. Currently, there are two types of CLE: (1) endoscopic-based or integrated confocal laser endomicroscope (eCLE or iCLE, Pentax, Tokyo, Japan) and (2) probe-based confocal laser endomicroscope (pCLE, Mauna Kea Technologies, Paris, France) [10, 11]. From various studies, CLE can provide real-time histology [12–19] and possibly dynamic cellular changing in gastrointestinal disease [20, 21]. Even though, the learning curve study in CLE is still limited. 

To our knowledge, this is the first study of the learning curve of GIM interpretation by both iCLE and pCLE. The acceptance of all new technologies requires not only high validity scores and good accessibility but also a short learning curve to attract practicing users. The previous study revealed the excellent sensitivity, specificity, and accuracy of a combination method with FICE and pCLE for GIM diagnosis at 94%, 85%, and 89%, respectively [4]. In addition, the other type of CLE called iCLE showed the near perfect sensitivity at 98% and specificity at 95% for GIM diagnosis based on the updated Sydney System's recommendation [22]. However, all interpretations of GIM by both CLE techniques were performed by skilful researchers. Lim et al. emphasized that experienced interpreters in iCLE reading can achieve greater validity score than the beginners in GIM diagnosis with the sensitivity of 95% versus 61% (*P* = 0.39) and specificity of 93% versus 62% (*P* < 0.001) [23]. Moreover, experienced iCLE interpretation provided almost perfect interobserver agreement (*κ* = 0.89), whereas the beginner showed only fair interobserver agreement (*κ* = 0.28) [23].

Because one of the important factors determining the good GIM reading outcome is the experience of interpreter, then it is important to study how difficult it is to train the beginners to become accurate in pCLE image interpretation. In our opinion, gaining the acceptable reading skill in GIM interpretation by pCLE is not too difficult because the endoscopic criteria for GIM reading are not complicate, and the goblet cells can be simply observed [22]. Our study confirmed this by showing that the interpretation of pCLE images in GIM can be learnt rapidly after a short training session and many of them achieved the high sensitivity of their interpretations within 2 sessions of the 4-session study format. Moreover, all of them can provide a very high PPV (≥80%) since the first test. It is emphasizing that the beginners can interpret GIM images correctly only after a brief learning period. In addition, almost all of the beginners can maintain the high interpretation skill with substantial (*κ* = 0.52) to almost perfect interobserver agreement (*κ* = 0.86).

**Figure 2:**
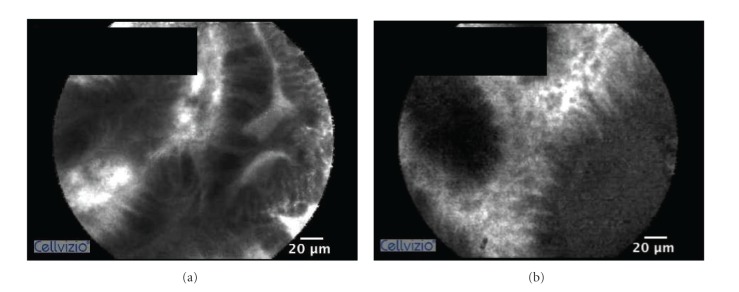
Comparing between high and poor quality pCLE imaging of gastric intestinal metaplasia. (a) High quality pCLE image shows clearly visualized epithelium and goblet cell. (b) Poor quality pCLE image shows unclear epithelial boarder and vessels.

However, the pCLE reading skill is not representing the pCLE endoscopic skill which is important to obtain the high quality images for interpretation. Achieving the skill to perform pCLE with high quality output may not be easy and need longer time to practice because of certain reasons such as the unfamiliarity of small fragile devices that need a gentle handling and the shaking effect of the pCLE image due to the very fine examination in a small area. Although, there has been no report on the effect of GIM image quality on the accuracy in GIM detection, a study of pCLE used to detect colonic neoplasm showed that the suboptimal image quality on pCLE resulted in a lower sensitivity of colonic adenoma detection [24]. Moreover, another recent study revealed that the interobserver agreement, sensitivity, specificity, and accuracy of distinguishing between neoplastic colonic polyp and nonneoplastic colonic polyp by pCLE were increased from 0.55, 76%, 72%, and 75% to 0.83, 88%, 89%, and 88%, respectively, after considering only good and excellent quality video clips [25]. Therefore, we may extrapolate that the quality of GIM image may be an important confounding factor affecting the difficulty of image interpretation. In our study selected only high quality video clips before we provided those to our trainees. All video clips in this study were chosen by our experienced endoscopist (RP) by using the criteria as follows: clearly visualized gastric epithelium, vessels, and goblet cell with no shaking effect or significant artifact. Although hyoscine injection is required to decrease gastric movement, it is not easy to obtain a high quality image by putting a tiny pCLE probe in the stomach that always has a lot of peristalsis. In our anecdotal experience, a significant period of training to stabilize a scope embedded with a transparent cap is also important to qualify a good MPEG stream. The examples of high and poor quality GIM images by pCLE are displayed in Figure 2.

To date there has been no study on the learning curve for GIM image interpretation by pCLE. However, there are a handful number on learning curve studies of CLE evaluating colonic polyp [5, 6] and inflammatory bowel disease (IBD) [7]. In those series, by post hoc assessment, they showed remarkable results. Kuiper et al. conducted a study to interpret 90 iCLE images of different colonic polyps including normal, regenerative, and neoplastic polyps. After a brief period of training session (30-image assessment), they found that the diagnostic accuracies by the 3 endoscopists were high (90%, 93%, and 93%, resp.). Moreover, the intra- and interobserver agreements were substantial (*κ* = 0.67 and 0.84, resp.) [5]. Additionally, Buchner et al. requested 11 endoscopists from different 3 centers to interpret the difference between 76 polyp images (neoplastic and nonneoplastic) obtained by pCLE. They found that the overall accuracy by 11 endoscopists was only 63% for the first 40 lesions; however, it gradually improved to 86% in the final 16 lesions. They concluded that accurate interpretation of pCLE images for predicting neoplastic colonic lesion can be learned rapidly by a wide range of GI specialists [6]. In the other aspect of colonic disease, Gaddam et al. showed the agreement in IBD mucosal readings between pCLE and histopathology improved over time with kappa values of 0.81 at the end of the study (twenty-six cases) [8]. Moreover, they collected the pCLE endoscopic skill information by using different performance parameters including total duration of the procedure, time to receive a pCLE image in focus, the ratio between total pCLE images obtained from each patient and the number of pCLE images in focus. They concluded that pCLE is easy to learn. However, the limitations of their study were only 2 blinded endoscopists included in the study and the absence of interobserver agreement analysis (Table 3). In addition, Gaddam et al. showed the substantial interobserver agreement (*κ* = 0.61) in both experienced and nonexperienced observers for Barrett esophagus diagnosis by pCLE [8]. In their study, they gave 1 hr and 10 video clips to 3 beginners during the formal training session. Then, 75 high quality pCLE video clips of dysplasia and nondysplasia in Barrett esophagus were evaluated in 2 parts, starting with the initial 30 pCLE video clips evaluation and followed by the other 45 pCLE video clips after the feedback session. They showed that the accuracies of readings on the first 30 (83%) and the last 45 video clips (81%) were not statistically significant (*P* = 0.51). 

There are certain limitations of the present study. The first was the lack of intraobserver agreement (test and retest validities on different beginners); therefore this study cannot show the consistency of each beginner for GIM evaluation by pCLE. Second, no external validation was assessed; therefore the result from this study may not be applicable to other centers that may have different types of beginners. Last, the result of this offline study cannot be extrapolated for the possible result of the real-time images interpretation.

In summary, the interpretation of GIM images obtained by pCLE can be learned rapidly after a short training period with at least substantial interobserver agreement. However, we cannot extrapolate the result of this study as pCLE examination for GIM diagnosis is easy to perform since the present study did not investigate the difficulty of pCLE scoping technique. And we also believe that skillful scoping technique is a requisite for the endoscopist to be able to obtain high quality pCLE images. 

## Figures and Tables

**Table 1:** Representing sensitivity, specificity, accuracy, PPV, and NPV in each beginner.

	Beginner number 1	Beginner number 2	Beginner number 3	Beginner number 4	Beginner number 5	*P* value
Sensitivity (%) (range)	87.5 (75, 100)	87.5 (75, 100)	97.2 (88.8, 100)	89.5 (81.8, 100)	90.9 (75, 100)	0.53
Specificity (%) (range)	75.2 (45.5, 88.8)	79.9 (66.6, 91.6)	85.6 (77.7, 91.6)	86.3 (81.8, 91.6)	75.2 (66.6, 81.8)	0.52
Accuracy (%) (range)	83.7 (70, 95)	86.2 (70, 95)	91.2 (85, 95)	87.5 (85, 90)	82.5 (70, 90)	0.57
PPV (%) (range)	94.3 (83.3, 100)	94.6 (80, 100)	97.4 (90, 100)	90.5 (80, 100)	91.7 (80, 100)	0.64
NVP (%) (range)	75.6 (60, 91.7)	79.1 (60, 91.7)	85.3 (80, 88.9)	84.2 (80, 87.5)	75.0 (60, 84.6)	0.55

**Table 2:** Representing accuracy, sensitivity, specificity, and Fleiss' kappa in overall assessment by the beginners.

	First test	Second test	Third test	Fourth test
Sensitivity (%) (range)	83.3 (75, 100)	96.3 (81.8, 100)	93.2 (88.8, 100)	97.5 (87.5, 100)
Specificity (%) (range)	71.6 (66.6, 91.6)	84.3 (77.7, 88.8)	72.7 (45.4, 81.8)	86.6 (75.0, 91.6)
Accuracy (%) (range)	81 (70, 95)	91 (85, 95)	82 (70, 85)	91 (85, 95)
Agreement (Fleiss' kappa)	0.44 (moderate)	0.82 (Almost perfect)	0.52 (Substantial)	0.86 (Almost perfect)

**Table 3:** Comparing the learning curve of different studies of CLE in the GI tract.

	Present study	Kuiper et al. Gastrointest Endosc 2012 [5]	Buchner et al. Gastrointest Endosc 2011 [6]	Neumann et al. Histol Histopathol 2011 [7]	Gaddam et al. Am J Gastroenterol 2011 [8]
Type of confocal laser	pCLE	iCLE	pCLE	pCLE	pCLE
Tissue	Stomach (GIM)	Colonic polyp	Colonic polyp	Colon (IBD)	Esophagus (Barrett)
Number of reader	5	3	11	2	3
Number of center	1	1	3	1	1
Type of study	Offline study	Offline study	Offline study	Offline study	Offline study
Training duration	1 hr	N/A	2 hr	N/A	1 hr
Intraobserver agreement (*κ*)	N/A	Yes (0.68–0.84)	N/A	N/A	N/A
Interobserver agreement (*κ*)	Yes (0.52–0.86)	Yes (0.67–0.73)	N/A	N/A	Yes (0.48–0.68)
Conclusion	Easy to learn	Easy to learn	Easy to learn	Easy to learn	Short learning curve

pCLE: probe-based confocal laser endomicroscope, iCLE: integrated confocal laser endomicroscope, GIM: gastric intestinal metaplasia, IBD: inflammatory bowel disease, and N/A: no data.

## References

[1] Ferlay J, Shin HR, Bray F, Forman D, Mathers C, Parkin DM (2010). Estimates of worldwide burden of cancer in 2008: GLOBOCAN 2008. *International Journal of Cancer*.

[2] Fox JG, Wang TC (2007). Inflammation, atrophy, and gastric cancer. *Journal of Clinical Investigation*.

[3] Correa P, Piazuelo MB, Wilson KT (2010). Pathology of gastric intestinal metaplasia: clinical implications. *The American Journal of Gastroenterology*.

[4] Pittayanon R, Rerknimitr R, Ridtitid W (2011). Magnified intelligence chromoendoscopy (FICE) plus probe-based confocal laser endomicroscopy (pCLE) for gastric intestinal metaplasia (GIM) diagnosis: a pilot feasibility trial. *Gastrointestinal Endoscopy*.

[5] Kuiper T, Kiesslich R, Ponsioen C, Fockens P, Dekker E (2012). The learning curve, accuracy, and interobserver agreement of endoscope-based confocal laser endomicroscopy for the differentiation of colorectal lesions. *Gastrointestinal Endoscopy*.

[6] Buchner AM, Gomez V, Heckman MG (2011). The learning curve of in vivo probe-based confocal laser endomicroscopy for prediction of colorectal neoplasia. *Gastrointestinal Endoscopy*.

[7] Neumann H, Vieth M, Atreya R, Neurath MF, Mudter J (2011). Prospective evaluation of the learning curve of confocal laser endomicroscopy in patients with IBD. *Histology and Histopathology*.

[8] Gaddam S, Mathur SC, Singh M (2011). Novel probe-based confocal laser endomicroscopy criteria and interobserver agreement for the detection of dysplasia in Barrett’s esophagus. *The American Journal of Gastroenterology*.

[9] Kiesslich R, Burg J, Vieth M (2004). Confocal laser endoscopy for diagnosing intraepithelial neoplasias and colorectal cancer in vivo. *Gastroenterology*.

[10] Meining A (2009). Confocal endomicroscopy. *Gastrointestinal Endoscopy Clinics of North America*.

[11] Kiesslich R, Canto MI (2009). Confocal laser endomicroscopy. *Gastrointestinal Endoscopy Clinics of North America*.

[12] Polglase AL, McLaren WJ, Skinner SA, Kiesslich R, Neurath MF, Delaney PM (2005). A fluorescence confocal endomicroscope for in vivo microscopy of the upper- and the lower-GI tract. *Gastrointestinal Endoscopy*.

[13] Hoffman A, Goetz M, Vieth M, Galle PR, Neurath MF, Klesslich R (2006). Confocal laser endomicroscopy: technical status and current indications. *Endoscopy*.

[14] Nguyen NQ, Leong RWL (2008). Current application of confocal endomicroscopy in gastrointestinal disorders. *Journal of Gastroenterology and Hepatology*.

[15] Xie XJ, Li CQ, Zuo XL (2011). Differentiation of colonic polyps by confocal laser endomicroscopy. *Endoscopy*.

[16] Li WB, Zuo XL, Li CQ (2011). Diagnostic value of confocal laser endomicroscopy for gastric superficial cancerous lesions. *Gut*.

[17] Dunbar KB, Okolo P, Montgomery E, Canto MI (2009). Confocal laser endomicroscopy in Barrett’s esophagus and endoscopically inapparent Barrett’s neoplasia: a prospective, randomized, double-blind, controlled, crossover trial. *Gastrointestinal Endoscopy*.

[18] Meining A, Chen YK, Pleskow D (2011). Direct visualization of indeterminate pancreaticobiliary strictures with probe-based confocal laser endomicroscopy: a multicenter experience. *Gastrointestinal Endoscopy*.

[19] Dunbar KB, Canto MI (2010). Confocal laser endomicroscopy in Barrett’s esophagus and endoscopically inapparent Barrett’s neoplasia: a prospective, randomized, double-blind, controlled, crossover trial. *Gastrointestinal Endoscopy*.

[20] Humphris J, Swartz D, Egan BJ, Leong RW (2012). Status of confocal laser endomicroscopy in gastrointestinal disease. *Tropical Gastroenterology*.

[21] Coron E, Mosnier JF, Ahluwalia A (2012). Colonic mucosal biopsies obtained during confocal endomicroscopy are pre-stained with fluorescein in vivo and are suitable for histologic evaluation. *Endoscopy*.

[22] Guo YT, Li YQ, Yu T (2008). Diagnosis of gastric intestinal metaplasia with confocal laser endomicroscopy in vivo: a prospective study. *Endoscopy*.

[23] Lim LG, Yeoh KG, Salto-Tellez M (2011). Experienced versus inexperienced confocal endoscopists in the diagnosis of gastric adenocarcinoma and intestinal metaplasia on confocal images. *Gastrointestinal Endoscopy*.

[24] Kuiper T, van den Broek FJ, van Eeden S, Fockens P, Dekker E (2012). Feasibility and accuracy of confocal endomicroscopy in comparison with narrow-band imaging and chromoendoscopy for the differentiation of colorectal lesions. *The American Journal of Gastroenterology*.

[25] Gómez V, Buchner AM, Dekker E (2010). Interobserver agreement and accuracy among international experts with probe-based confocal laser endomicroscopy in predicting colorectal neoplasia. *Endoscopy*.

